# Impact of Patients, Nurses, and Workload on the Use of a Nurse-Initiated Pain Protocol at Triage in the Emergency Department: A Single-Center Retrospective Observational Study

**DOI:** 10.3390/jcm15020782

**Published:** 2026-01-18

**Authors:** Yvan Fournier, Patrick Taffe, Corrado Corradi-Dell’Acqua, Olivier Hugli

**Affiliations:** 1 Emergency Department, Hôpital Intercantonal de la Broye, 3 Avenue de la Colline, 1530 Payerne, Switzerland; yvan.fournier@hibroye.ch; 2 Center for Primary Care and Public Health (Unisanté), University of Lausanne, 44 Rue du Bugnon, 1011 Lausanne, Switzerland; patrick.taffe@unisante.ch; 3 Center for Mind/Brain Sciences (CIMeC), University of Trento, Via delle Regole, 101, 38123 Mattarello, Trento, Italy; corrado.corradi@unitn.it; 4 Faculty of Psychology and Educational Sciences, University of Geneva, 101 Boulevard Carl Vogt, 1205 Geneva, Switzerland; 5 Emergency Department, Lausanne University Hospital, Lausanne University, Bugnon 46, 1011 Lausanne, Switzerland

**Keywords:** nurse-initiated pain protocol, emergency department, workload, triage operational context, triage, analgesia

## Abstract

**Background**: Nurse-initiated pain protocols (NIPPs) at emergency department (ED) triage remain underused. This study investigated factors associated with patient refusal and nurse use of NIPP, accounting for triage operational context. **Methods**: This retrospective observational study combined prospectively collected nurse characteristics with retrospective data on NIPP use over 15 months in a tertiary university hospital ED. Outcomes included rates of NIPP refusal and use, documented reasons for refusal, and associations with patient characteristics, nurse characteristics, crowding, and operational pressure. **Results**: Sixty-three triage nurses managed 16,137 adult patients; 6.2% refused the NIPP. Among consenting patients, NIPP was used in one-third of encounters. Multi-level logistic regression revealed significant variation between nurses in both refusal and use. Refusal was more likely in patients with lower acuity and among nurses trained in Europe or concerned about prescribing responsibility, but less frequent with severe pain or longer triage duration. NIPP use was more frequent with lower acuity, higher pain intensity, longer triage duration, crowding, and among nurses with European training, but decreased in older patients and those arriving by ambulance. **Conclusions**: NIPP refusal and use at triage were both low, with marked variability between nurses. Patient characteristics and triage operational factors were most strongly associated with outcomes, while nurse-related factors contributed less. These findings support prospective implementation studies to clarify drivers of practice variation and optimize analgesia delivery at triage.

## 1. Introduction

Nurses and doctors in emergency departments (EDs) continually care for patients in pain as up to 80% report such a symptom of at least moderate severity [1,2]. However, despite improvement in management strategies over recent decades, pain is still insufficiently treated in the ED, a phenomenon known as oligoanalgesia [2]. Only one-half of patients receive analgesics and the door-to-analgesia wait is generally over 60 min [3]. To improve healthcare quality and counteract oligoanalgesia, EDs have implemented nurse-initiated pain protocols (NIPPs), which allow for initial pain management at triage or in the waiting room prior to consultation with a physician [4].

Despite the benefits of NIPPs, their use in daily practice faces multiple barriers related to organizational or resource-related issues, as well as nurses’ or patients’ idiosyncratic characteristics [5,6,7]. For example, nurses vary significantly in the degree to which they use NIPPs, a practice variation partially explainable by their personality [5,8]. Similarly, patients vary extensively in their perception of nurse-led analgesia, with the rate of NIPP refusal varying from 22% to 49% [9,10]. Patients base their refusal on a tolerable pain level, self-administration of analgesics prior to the ED visit, concerns about analgesic side effects or potential drug interactions with their usual medication, and even the risk of addiction to analgesics.

Pain assessment and treatment constitute social transactions, influenced by both clinical characteristics and the personal attributes of patients and healthcare providers [8,11]. For instance, pain sensitivity is influenced by interpersonal factors that regulate social interactions, such as conformity, attraction, and group membership [12,13,14]. Accordingly, the transaction between patients and healthcare providers could shape providers’ judgment regarding the validity of pain complaints, which in turn can influence the decision to use NIPPs. Furthermore, crowding may reduce the likelihood of NIPP use [15].

We therefore distinguished refusal (acceptability of an offered intervention) from non-use despite eligibility (adoption in care delivery), consistent with established implementation-outcome frameworks [16]. Prior ED studies describe analgesic refusal and its determinants [9,17], and separately document incomplete adherence to pain protocols [5,18], supporting these as distinct phenomena. However, the determinants of NIPP refusal and NIPP use remain poorly understood, particularly the relative contributions of patient- and nurse-level characteristics and the role of crowding and operational pressure at triage. A better understanding of these determinants is an essential first step toward developing strategies to improve NIPP implementation at triage [19]. The aims of the present study were therefore to examine rates of patient refusal and nurse use of our NIPP, as well as their association with patient characteristics, nurse characteristics, and triage operational factors at the time of triage.

## 2. Methods

### 2.1. Study Design

We conducted a monocentric retrospective observational study at a large Swiss university hospital ED (~45,000 annual visits) [5]. The NIPP is shown in Figure S1.

### 2.2. Sample and Population

Eligible patients were those triaged by a nurse certified to use the NIPP, had a documented pain by numeric rating scale (NRS) > 0, and no contraindication to use of the NIPP or its included analgesics. Exclusion criteria were life-threatening emergency (Swiss Emergency Triage Scale level 1) [20], chronic pain, intoxication, or age < 16 years. Chronic pain was defined as pain lasting > 3 months and was identified using a dedicated checkbox completed at the index ED visit by the triage nurse in our custom, in-house triage software developed and maintained by our emergency department. Suspected intoxicat.ion (alcohol and/or drugs) was similarly identified using a triage checkbox. Patients were classified into three groups: (1) NIPP refusal; (2) NIPP used; and (3) NIPP not used despite eligibility.

### 2.3. Data Collection, Variables, and Measures

Triage data is entered in our flux software and stored locally on a secure drive from which data was extracted, ensuring standardized data collection. Patient data included their demographics, mode of arrival (self-referral vs. ambulance transport), triage severity according to the Swiss Emergency Triage Scale [20], pain intensity using a numerating rating scale bounded by 0 (no pain) to 10 (worst pain imaginable), orientation within the ED, and finally if and why patients refused the NIPP. Triage duration was calculated as the time from the initiation timestamp to the closure timestamp and was considered a marker of triage process/interaction time (which may be influenced by NIPP-related activities as well as other clinical and communication needs). Front-end crowding, or operational pressure, was approximated by the waiting room census at the time of triage (patients awaiting triage plus triaged patients awaiting transfer), an objective, routinely collected indicator.

Nurse survey collected data on sociodemographic characteristics, personality, work experience, personal experience with pain, and perceived enablers and barriers to using the NIPP. Stress from decision-making under uncertainty was assessed using the Stress from Uncertainty Scale (SUS) and their attitude toward risk-taking using the Risk-Taking Scale [21,22]. Nurses who did not participate did not provide informed consent; therefore, no individual-level data was collected for non-responders and responder–non-responder comparisons were not possible.

### 2.4. Outcomes

The two primary outcomes were (1) the percentage of patients who refused the NIPP at triage; and (2) the proportion of eligible patients who received the NIPP at triage. Refusal was defined as documentation that NIPP was proposed and declined. Non-use despite eligibility was defined as absence of NIPP administration among eligible patients without documented refusal. Secondary outcomes were to quantify the between-nurse variations in these outcomes and to assess the relative contribution of patient characteristics, nurse characteristics, and triage operational context (front-end crowding and triage process time).

### 2.5. Statistical Analysis

Nurse, patient, and triage operational characteristics were summarized using descriptive statistics, reported as means ± standard deviation (SD) or medians and interquartile range [IQR]. Group comparisons were performed using Student’s *t*-test or the Wilcoxon–Mann–Whitney test for continuous variables, and the Chi-squared test or Fisher’s exact for categorical variables, as appropriate.

Given the hierarchical structure of our data, with ED patients (level 1) nested within triage nurses (level 2), a random-intercept multi-level logistic model was applied to account for the intra-nurse clustering of observations [23,24]. Nurse-level (level 2) covariates were available only for survey respondents; accordingly, multi-level models including level 2 variables were fitted using encounters triaged by participating nurses. Three models were estimated to assess the contribution of covariates at each level to the reduction in residual heterogeneity (i.e., contextual effect after including covariates): (1) an empty model including only a random intercept (null model); (2) the null model plus level 1 variables (patient characteristics; model 1); and (3) a model including both level 1 and level 2 variables (patient and nurse characteristics; model 2). To separate within-cluster and between-cluster effects of patient (level 1) variables and reduce confounding by cluster, each variable was decomposed into within-cluster and between-cluster components [25]. Because triage duration may be partly influenced by NIPP administration, we conducted a sensitivity analysis excluding triage duration from the multi-level models.

Missing data was not imputed. For each outcome, the three nested multi-level models were fitted using a complete-case dataset (i.e., encounters with no missing values for any variable included in the full model), to enable direct comparison across models. Nurses with no complete-case encounters for a given outcome were not retained in the corresponding multi-level analyses, reducing their number from 63 to 58.

Interpretation of model coefficients, quantification of the residual contextual effect (i.e., the effect of nurse clusters on the outcome after accounting for nurse-level variables) using the median odds ratio (MOR), and assessment of associations between nurse characteristics (contextual variables and outcomes using the interval odds ratio (IOR)) are presented in Supplementary S1 [26,27,28,29].

Estimation was performed using maximum likelihood and odds ratios (ORs) with 95% confidence intervals (CIs) are reported. A bilateral *p*-value < 0.05 was considered statistically significant. All analyses were conducted using Stata, version 18 (StataCorp, College Station, TX, USA).

### 2.6. Artificial Intelligence

ChatGPT (OpenAI) was used solely for language editing (grammar, phrasing, and fluency). It was not used to generate scientific content, interpret data, or draw conclusions. All changes were verified by the authors.

## 3. Results

### 3.1. Patient Characteristics (Table 1)

**Figure 1:**
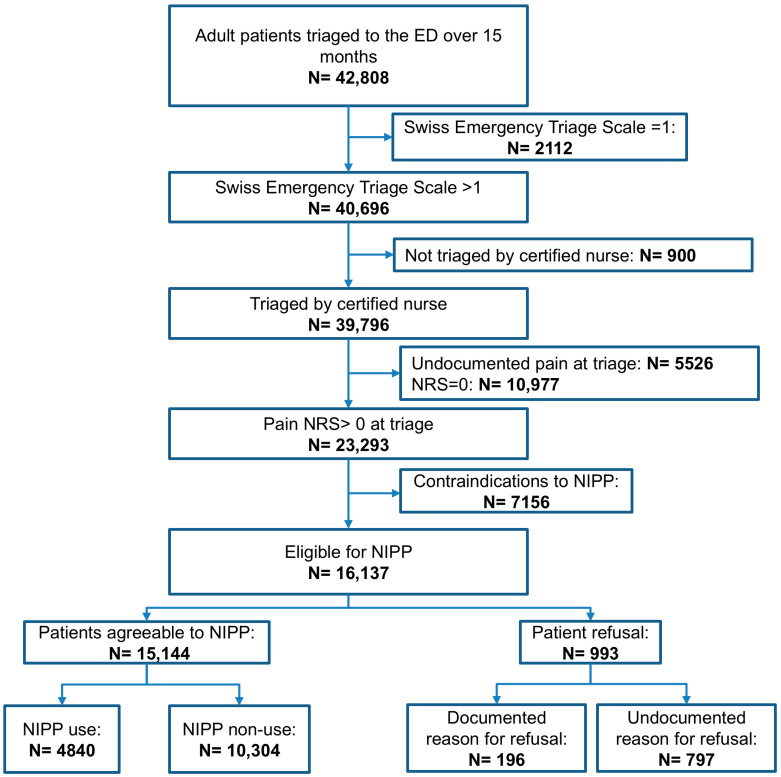
Patient flow chart. NRS = numeric rating scale; NIPP = nurse-initiated pain protocol.

Of 42,808 triaged patients over 15 months, 23,293 (54.4%) had a documented numeric pain rating scale > 0 in the electronic health record (Figure 1), of which 16,137 (37.7%) were eligible for the NIPP. Among them, 993 (6.2%) refused the NIPP; 4840 (32%) received; and 10,304 (68%) did not despite eligibility.

**Table 1:** Use, refusal, and non-use of the nurse-initiated pain protocol (NIPP) according to patient characteristics.

Variable	All Patients N = 16,137	Refusal of NIPP N = 993	Use of NIPP N = 4840	No Use of NIPP N = 10,304	*p*-Value
Gender, n (%)					0.338
Male	8606 (53.7)	555 (55.9)	2585 (53.4)	5517 (53.5)	
Female	7425 (46.3)	438 (44.1)	2255(46.6)	4787 (46.5)	
Median age, years (IQR)	40 (27–59)	36 (25–53)	35 (25–49)	44 (29–66)	<0.001
Admission route, n (%)					<0.001
Ambulance	2646 (16.4)	87 (8.8)	242 (5.0)	2317 (22.5)	
Outpatient	13,491 (83.6)	906 (91.2)	4598 (95.0)	7987 (77.5)	
Triage category SETS, n (%)					0.05
2	2090 (13.0)	54 (5.4)	325 (6.7)	1711 (16.6)	
3	11,129 (69.0)	728 (73.3)	3695 (76.3)	6706 (65.1)	
4	2918 (18.0)	211 (21.3)	820 (17.0)	1887 (18.3)	
Triage time, minute (IQR)	5 (4–8)	5 (3–7)	6 (4–8)	5 (4–7)	<0.001
Patients in waiting room, n (IQR)	6 (4–10)	6 (4–10)	6 (4–10)	6 (3–9)	0.001
NRS pain score, unit (SD)	5.3 (2.5)	5.0 (2.1)	6.9 (2.0)	4.5 (2.4)	<0.001
ED orientation after triage, n (%)					<0.001
Major medical/trauma	2848 (17.7)	50 (5.0)	292 (6.0)	2506 (24.3)	
Minor medical/trauma	13,289 (82.3)	943 (95.0)	4548 (94.0)	7798 (75.7)	

IQR = interquartile range; SETS = Swiss Emergency Triage Scale; NRS = numeric rating scale; SD = standard deviation; ED = emergency department.

Patients who refused the NIPP or did not receive NIPP were younger, less often brought by ambulance, and more frequently triaged as category 2. Patients who received NIPP reported higher pain scores on admission. NIPP use was less frequent for patients assigned to the major medical trauma area, and the number of patients in the waiting room was slightly lower when NIPP was used.

Reasons for NIPP refusal were documented in 199 of 993 patients (20.0%); the following distribution therefore pertains to the documented subset: bearable pain (43.7%), preference to wait for physician assessment (17.6%), fear of analgesic side effects (7.5%), refusal of the proposed analgesic (7.0%), perceived inefficacy of the proposed analgesic (5.1%), and refusal of oral administration (4.5%) (Table 2).

### 3.2. Nurse Characteristics (Table 3)

Sixty-three (67.7%) certified triage nurses completed the survey (mean age, 34.8 years). Most were female, employed at a high full-time equivalent, and had on average 7.3 years of ED experience. More than 60% had received some form of analgesia training. The mean duration of NIPP use before data collection was 233 days.

Nearly 80% of nurses reported personal experience of severe pain, most often traumatic low back pain or headache. Nurses estimated using the NIPP in ~70% of eligible patients, whereas actual use was below one-third. Fewer than 20% perceived the autonomous administration of analgesics under the NIPP as burdensome.

**Figure 2:**
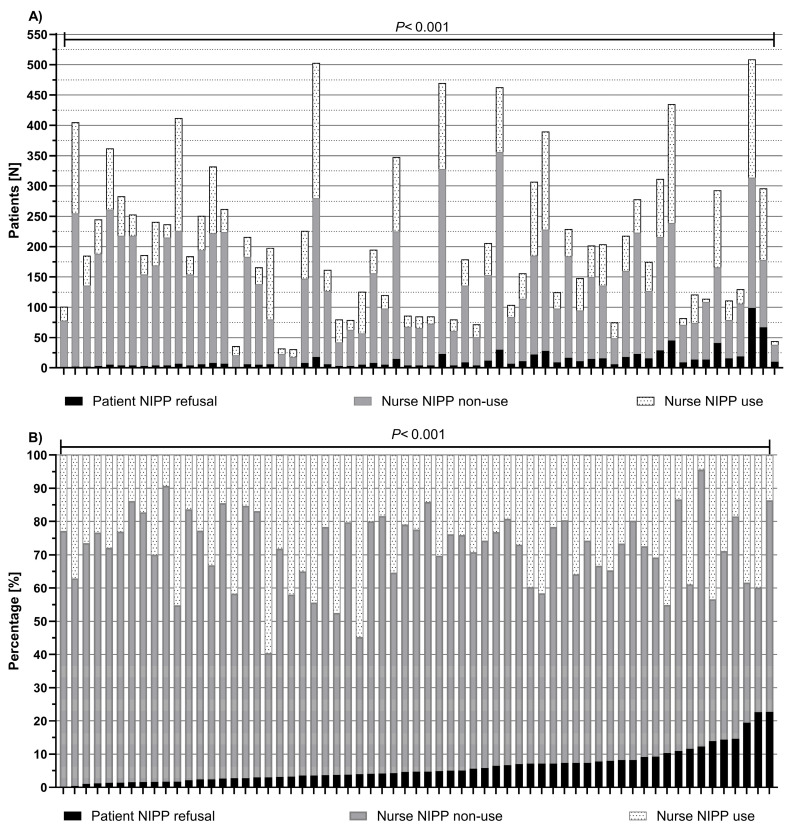
Distribution of nurse-initiated pain protocol (NIPP) use, non-use despite eligibility, and patient refusal at triage across nurses (N = 63): (**A**) absolute counts; (**B**) percentage.

The median number of triages per nurse was 195 (IQR 111–283), with a median of 7 NIPP refusals (4.7%). Both refusal and NIPP use varied significantly across nurses (*p* < 0.001) (Figure 2).

**Table 3:** Demographic, professional, and personal and characteristics of triage nurses.

Characteristics (N = 63)	
Age, year [SD]	34.8 (7.0)
Female, n (%)	45 (71.4)
Full-time equivalent *, % [SD]	91.4 [15.9]
Place of training, n (%)	
Switzerland	25 (39.7)
European Union	28 (44.4)
Canada	10 (15.9)
Certification in emergency care, n (%)	11 (17.5)
Years since graduation, years (IQR)	9 (6–12)
ED experience, years (IQR)	6 (4–9)
Specific pain management training **, n (%)	38 (61.3)
Personal experience of severe pain, n (%)	50 (79.4)
Cause of severe pain, n (%):	
Traumatic	27 (54.0)
Low-back	23 (50.0)
Headache	22 (47.8)
Abdominal	19 (41.3)
Childbirth	15 (32.6)
Renal colic	10 (21.3)
Neuropathic	5 (10.9)
Number of different causes of severe pain, n (IQR)	2 (1–3)
Risk-Taking Scale score, points [SD]	15.7 [2.7]
Stress from Uncertainty Scale, points [SD]	19.8 [5.3]
Burden of prescribing NIPP experienced as heavy, n (%)	11 (17.7)
Experience with NIPP;	
Previous experience with another NIPP, n (%)	15 (24.2)
Duration of NIPP use, days [SD]	233 [64]
Number of triages per nurse during the study period, n (IQR)	195 (111–283)
Percentage use of the NIPP, % (IQR):	
Estimated	75 (50–80)
Actual	26.5 (19.8–35.8)
Fear to administer analgesia, n (%):	
Acetaminophen	3 (4.8)
Ibuprofen	4 (6.4)
Tramadol	7 (11.1)
Patient NIPP refusal	
n (IQR)	7 (4–16)
% (IQR)	4.7 (2.8–7.8)

* Minimal full-time equivalent = 50%. ** Provided by the ED. NIPP = nurse-initiated pain protocol; IQR = interquartile range; SD = standard deviation.

### 3.3. Multi-Level Logistic Regression

**Figure 3:**
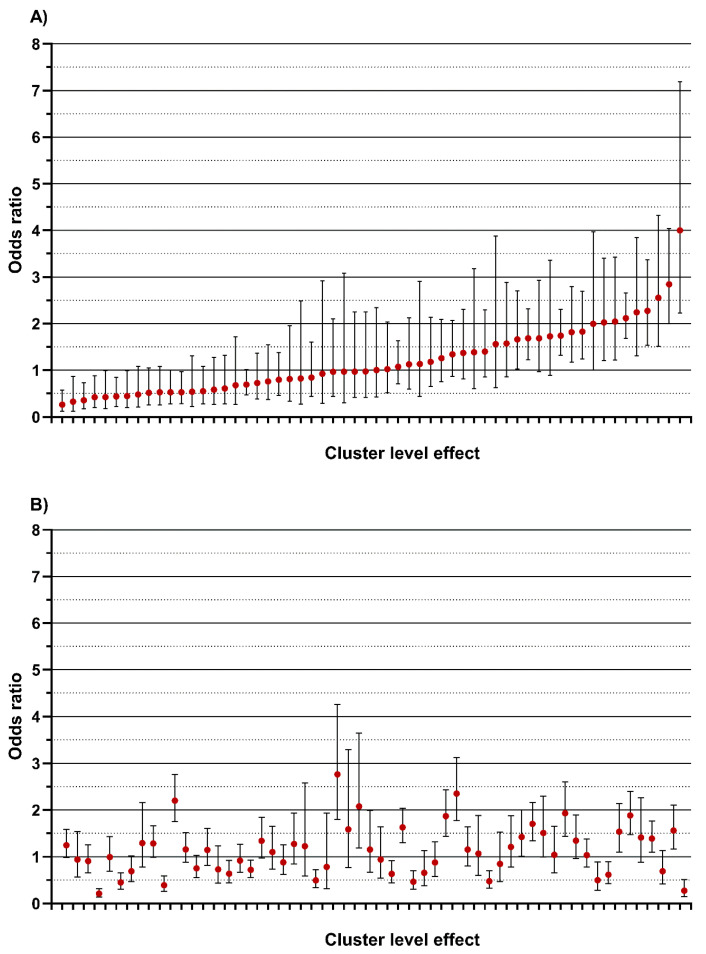
Comparison of each triage nurse with an “average triage nurse” (N = 58 nurses (clusters) with ≥1 complete-case encounter included) after adjustment for observed patient, nurse characteristics, triage process, and crowding regarding: (**A**) patient NIPP refusal; (**B**) nurse NIPP use. NIPP = nurse-initiated pain protocol. Each point represents one triage nurse. The horizontal line at OR = 1 represents the average nurse. Nurse with an OR > 1 have higher odds of patient NIPP refusal (**A**) or NIPP use (**B**) than the average nurse for otherwise similar patients, while those with OR < 1 have lower odds. The whiskers are 95% CIs: if a nurse’s CI does not cross 1, their odds are statistically different from the average nurse; if it crosses 1, the data are compatible with no difference from average.

For NIPP refusal (Table 4), the empty model MOR was 2.15, indicating significant variation between nurses. Including patient-level variables (level 1) showed that NIPP refusal was less likely for patients transported by ambulance, with a lower triage severity, more severe pain, or longer triage duration. Adding nurse-level variables (level 2) revealed that only European training and perceived burden of NIPP prescription was associated with increased refusal. Cluster-level ORs varied significantly between nurses (Figure 3A). The MOR decreased slightly to 1.95 after adding level 2 variables. The IORs for nurse characteristics were wide, consistently including 1, indicating substantial residual heterogeneity between clusters.

For NIPP use (Table 5), the empty model MOR was 1.71. Patient-level variables indicated lower likelihood of NIPP use for older patients, those transported by ambulance, or with lower triage severity, and higher likelihood for patients with more severe pain, longer triage duration, or higher waiting room occupancy. Only European training among nurses was positively associated with NIPP use. In the full model, the MOR remained similar to the null model.

Even after adjusting for observed patient characteristics, nurse characteristics, triage process, and crowding variables, there remains meaningful between-nurse variability in both outcomes: some nurses had systematically higher/lower odds of refusal (Figure 3A) and higher/lower odds of NIPP use (Figure 3B) compared with the “average nurse”. In sensitivity analyses excluding triage duration in both models, the estimates for the other covariates were material unchanged.

## 4. Discussion

The aim of this study was to quantify patient refusal rates of a NIPP at triage, evaluate NIPP use by triage nurses, and identify patient, nurse, and operational factors associated with these outcomes. Although NIPP has been a proven approach to provide timely, safe, and effective acute pain relief in the ED [4], our results indicate that many eligible patients did not receive NIPP at triage and three main findings emerged. First, a small but non-negligible proportion of patients refused the protocol but reasons for refusal were documented in only 199 of 993 refusals (20.0%), highlighting substantial missingness in triage documentation. Second, even in the absence of refusal, triage nurses applied the NIPP in only one-third of eligible patients, a low proportion consistent with prior studies [19]. Finally, there were significant between-nurse variations in both refusal or use of the NIPP, both partly associated with patient and nurse characteristics but also by contextual factors. Together, our findings provide new insights into real-life NIPP use at triage.

### 4.1. Patient Characteristics

This study includes the largest sample to date analyzing patient refusals, with nearly 16,000 eligible patients. Our results align with the existing literature: patients who refuse analgesia tend to be younger, self-referred, have a lower triage acuity, report less intense pain, and are typically managed in minor ED areas [9,30,31]. In this cohort, the refusal rate was around 6.2%, lower than the 15–49% reported elsewhere [30,32]. This discrepancy may reflect different patients’ attitudes towards pain management, or methodological factors such as prospective study designs that capture refusals more systematically.

Among documented refusals (20% of refusals), one in six were due to objections to the specific analgesic, its perceived inefficacy, or the oral route of administration. Our NIPP relies exclusively on orally administered analgesics, which may not match patient expectations. Expanding the protocol to include other molecules (e.g., strong opioids, methoxyflurane) and alternative routes (intranasal, intramuscular, intravenous) may reduce refusal [33,34]. Incorporating shared decision-making regarding analgesics choice may further improve acceptance [34,35].

Older patients were less likely to refuse, but paradoxically also less likely to receive NIPP, as reported by others [19]. Their pain assessment is more complex due to reporting difficulties, comorbidities, and higher risk of adverse effects [36]. Ambulance-transported patients, although rare in our sample, were less likely to receive NIPP, possibly because they had already received prehospital analgesia (including opioids) under paramedic-led pain protocols or because they were transferred rapidly to treatment bays [19]. In contrast, patients with higher pain scores, longer triage times, or higher triage acuity were more likely to receive NIPP, reflecting clinical urgency [19], which has been shown experimentally to trigger a greater sense of care and motivation to help patients [37]. While race and ethnicity are known to influence triage decisions and analgesia provision [38,39], these data are not routinely collected in Switzerland and could not be accounted for in our analyses.

### 4.2. External Factors and System Barriers

Higher waiting room occupancy was not associated with NIPP refusal but with a slightly higher likelihood of NIPP use. This measure reflects front-end crowding and queuing—and therefore anticipated waiting times—rather than a direct measure of individual nurse activity at triage. Because triage staffing levels were not available retrospectively, this association should be interpreted cautiously and may be confounded by unmeasured operational factors (including potential staffing adjustments). Nevertheless, this contrasts with findings in ED treatment areas, where overcrowding has been associated with undertreatment of pain in some [40], but not all, studies [41]. One possible explanation is that triage occurs early in the care pathway and involves a one-to-one nurse–patient interaction. In this context, crowding may signal longer expected delays to physician assessment and thereby encourage nurses to initiate analgesia at triage [42]. As reported by others, a longer triage time was associated with lower odds of refusal and higher odds of NIPP use, which may reflect additional time available for communication and shared decision-making [31].

### 4.3. Triage Nurse Characteristics

Variation between nurses (median odds ratios or MOR > 1) contributed to both patient refusal and NIPP use. From a practical perspective, the MOR translates residual between-nurse heterogeneity onto the same scale as fixed-effect odds ratios. For refusal, an MOR of 2.15 in the null model (and 1.95 after adjustment) means that, for two otherwise identical patients, the median increase in the odds of refusal when triaged by a nurse with higher versus lower propensity to refusal is approximately two-fold. Given an overall refusal rate of 6.2% in our cohort, a two-fold difference in odds corresponds approximately to a change in refusal probability from ~6% to ~11% for otherwise comparable patients, illustrating a clinically meaningful nurse-related component. For NIPP use, the MOR was ~1.7, suggesting that between-nurse differences can also meaningfully affect protocol delivery even after accounting for observed covariates. These findings support the relevance of standardization and implementation strategies (e.g., audit and feedback, workflow support, and targeted training) to reduce unwarranted practice variation [43,44].

Although nurses had been positive about using the NIPP in their work [45], one in five perceived autonomous prescription as a burden, which was associated with higher NIPP refusal. This suggests that refusal is not entirely independent of nurse attitudes, supporting the view that pain management is a social transaction shaped by both patient and provider [8]. Other measured nurse-related factors (age, gender, operational context, personal pain experience, empathy, or risk-taking attitude) were not significantly associated with NIPP refusal and use.

Education in the European Union was consistently associated with greater odds of both refusal and use, although the underlying reasons remain unclear. Differences in pain management training, cultural norms, or communication styles may explain this finding [46,47]. However, the IOR for European country of training (0.552–4.207 for NIPP use) crossed 1, as did the IORs for all other level 2 covariates, suggesting that these measured nurse-level characteristics do not consistently explain the observed between-nurse variability and that residual nurse-to-nurse heterogeneity outweighs any uniform effect of training location. The persistence of residual heterogeneity suggests that unmeasured factors may influence both outcomes. These may include communication style, empathy, implicit bias, and patient nonverbal cues (e.g., behavior or facial expressions) that can shape decisions about analgesia [5,8,31,48,49]. Perceived time pressure and other environmental pressures at triage may also influence whether time-consuming interventions such as NIPP are offered and delivered [50,51]. These hypotheses warrant prospective investigation.

It could be hypothesized that nearly 80% of triage nurses who had experienced severe pain themselves would demonstrate lower rates of patient refusal or higher rates of NIPP use. However, experimental data indicate that the observers’ personal pain experience does not necessarily improve their ability to accurately detect pain in others [52]. Uncertainty is also known to influence decision-making at triage [53]. In a previous analysis of our data, nurses with higher SUS score documented more frequent contraindications to NIPP use [5], yet the SUS was not associated here with either patients’ NIPP refusal or nurses’ NIPP use among eligible patients. Similarly, the Risk-Taking Scale showed no significant association with NIPP use, in contrast to prior findings among emergency physicians [15].

### 4.4. Strength and Limitations

Our study has several strengths. First, the use of routine data, at a time when nurses were unaware of the study, minimized the risk of a Hawthorne effect that could have altered their pain management practices [54]. Second, our analysis included almost 200 triage encounters per nurse, likely capturing a broad spectrum of patient characteristics typically encountered in the ED. Third, the use of multi-level modeling allowed us to account for the natural clustering of patients within nurses, and to disentangle the respective contributions of patient characteristics, nurse characteristics, and front-end crowding and operational pressure at the time of decision-making.

This study also has limitations. First, reasons for refusal were missing in nearly 80% of cases. Because documentation of refusal reasons was discretionary, missingness is likely not random. Reasons may therefore be over-represented among encounters with more extensive nurse documentation or more complex discussions, whereas brief refusals or reasons perceived as self-evident may be under-documented; accordingly, refusal motives should be interpreted as descriptive for the documented subset rather than representative of all refusals. The documented reasons are broadly similar to those reported in prior ED studies, and NIPP included tolerable pain levels, a preference to wait for medical consultation, and concerns about analgesic side effects [9,17,31,55]. Second, triage operational factor was approximated using waiting room census. This proxy does not directly quantify individual nurse activity, particularly when staffing varies dynamically. Because staffing levels at triage were not available retrospectively, we could not compute nurse-to-patient ratios or adjust for real-time triage staffing. Third, our analyses were based on questionnaires completed by 68% of triage nurses. Although this represents a majority, no data were available for non-responders; we could not assess responder–non-responder differences; survey non-response may therefore introduce selection bias and limit generalizability of nurse-level associations. Moreover, the participating nurses were predominantly of European origin, which limited the diversity of cultural and ethnic backgrounds known to influence clinical practice [56,57]. Fourth, triage duration may be partly downstream of NIPP delivery (e.g., information, consent, dispensing) and therefore may act as a mediator rather than a purely exogenous predictor; we therefore interpret its association with NIPP refusal/use cautiously; however, removing triage duration from the model did not materially change the direction or magnitude of the other covariate estimates. Fifth, the study was monocentric, conducted in the ED of a tertiary care hospital that also functions as the city’s primary hospital, which may restrict the generalizability of our findings. Therefore, transferability of these findings should be considered in light of local context. Baseline NIPP use or refusal rates and the strength of operational associations may be context-specific, depending on triage organization, the scope of nursing autonomy to initiate analgesia, local eligibility criteria and analgesic formulary, and documentation workflows. Nevertheless, several insights are likely to generalize across EDs: distinguishing refusal from non-use despite eligibility, the contribution of patient-level characteristics to both outcomes, and the presence of meaningful between-nurse variability. Sixth, chronic pain and intoxication were identified using triage software checkboxes completed during routine care; therefore, misclassification is possible if these conditions were present but not recorded or conversely recorded when not present. Finally, as in any observational study, causal inferences between variables and outcomes cannot be drawn.

### 4.5. Implications for Practice

NIPPs are still underused at triage and varied substantially across triage nurses. Even after adjustment for measured patient and nurse characteristics and triage operational context (including crowding and time pressure), protocol use and refusal differed meaningfully between nurses, as reflected by MOR greater than 1; in addition, the nurse-level factors we measured did not consistently explain this variation, with wide IOR including one; these findings support further work to identify modifiable sources of practice variation and to evaluate system-oriented implementation approaches (e.g., standardization, workflow support, audit and feedback, and targeted training). In our observational analyses, patient characteristics and triage operational context showed stronger associations with refusal and/or NIPP use than measured nurse characteristics, suggesting that organizational factors may be important levers for improving protocol uptake. For example, interventions aimed at supporting timely decision-making under operational pressure warrant prospective evaluation. In addition, increased awareness of patient factors associated with refusal, while acknowledging that refusal motives were incompletely documented, may help tailor communication and shared decision-making when analgesics included in NIPPs are offered. Overall, these results generate testable hypotheses that future prospective or implementation studies can assess to optimize pain care at triage.

## 5. Conclusions

This study showed substantial between-nurse variability in NIPP refusal and use. Refusal was mainly associated with patient characteristics, whereas NIPP use was associated with patient characteristics and triage operational context (waiting room occupancy as a proxy for crowding). Residual between-nurse heterogeneity suggests unmeasured determinants. Future prospective studies should investigate these to optimize analgesia delivery at triage.

## Figures and Tables

**Table 2:** Documented reasons for patient refusal of the nurse-initiated pain protocol (NIPP).

Reasons Reported by Patients (N = 199)	Frequency, n (%)
Bearable pain	87 (43.7)
Waiting for medical consultation and diagnosis	35 (17.6)
Fear of side effects, drug interactions, or intolerance	15 (7.5)
Refusal of the specific medication	14 (7)
Inefficacy of the proposed analgesic	10 (5.1)
Refusal of oral administration	9 (4.5)
Other reasons	29 (14.6)

**Table 4:** Multi-level logistic regression for patients’ refusal of nurse-initiated pain protocol (NIPP) (N = 58).

Variables, OR ^#^ (95% CI)	Null Model	Model with Level 1 Variables, OR (95% CI)	Model with Level 1 and Level 2 Variables, OR (95% CI)
Fixed-effects variables, varying within cluster			
		Patient characteristics	
Gender Male Female		Reference 1.005 (0.860–1.174)	Reference 1.005 (0.860–1.175)
Age, year		0.997 (0.979–1.001)	0.997 (0.992–1.001)
Mode of transportation: Self-referral By ambulance		Reference 0.505 (0.379–0.673) **	Reference 0.504 (0.378–0.672) **
Triage severity scale:			
2		Reference	Reference
3		2.743 (1.885–3.992) **	2.738 (1.881–3.985) **
4		3.072 (2.035–4.638) **	3.068 (2.032–4.631) **
Pain by numeric rating scale, unit		0.937 (0.908–0.967) **	0.937 (0.908–0.967) **
		Triage process and crowding	
Triage duration in minutes		0.924 (0.908–0.967) **	0.923 (0.894–0.953) **
Patients in waiting room, n		1.007 (0.990–1.025)	1.007 (0.989–1.025)
		Nurse characteristics	
Gender Male Female			Reference 1.173 (0.713–1.932)
Age, year			1.012 (0.936–1.093)
Postgraduate experience			0.950 (0.841–1.073)
ED experience			1.001 (0.897–3.134)
Country of training Switzerland/Canada European Union			Reference 1.751 (1.057–2.902) *
Risk-taking scale, point			1.067 (0.974–1.166)
Stress from Uncertainty Scale, points			0.961 (0.916–1.008)
Burden of prescribing responsibility No Yes			Reference 2.003 (1.109–3.616) *
Number of different causes of severe pain, n (range, 0–6)			1.047 (0.886–1.238)
Fixed-effects variables, constant within cluster, IOR ^Φ^ (95%CI)			
Nurse gender Male Female			Reference 0.331–4.165
Nurses’ age			0.285–3.591
Certification in emergency care No Yes			Reference 0.464–5.852
Country of training Switzerland/Canada European Union			Reference 0.493–6.216
Postgraduate experience			0.268–3.371
ED experience			0.300–3.784
Risk-Taking Scale			0.276–3.943
Stress from Uncertainty Scale			0.271–3.411
Burden of prescribing responsibility No Yes			Reference 0.564–7.110
Number of painful body areas			0.295–3.717
Random effects Nurse’s MOR ^Σ^	2.15	2.15	1.95
Log likelihood	−2632.2	−5237.8	−2546.8
N	11,978 ^¶^	11,978 ^¶^	11,978 ^¶^
Likelihood-ratio test	-	<0.001	<0.001

* *p* < 0.05; ** *p* < 0.001. ^#^ OR = odds ratios; ^Φ^ IOR = interval odds ratios; ^Σ^ MOR = median odds ratios; CI = confidence interval. Logistic regression adjusted for means of level 1 variables. ^¶^ Complete-case analysis; N refers to encounters with complete data for all variables in this model.

**Table 5:** Multi-level logistic regression for nurses’ use of the nurse-initiated pain protocol (NIPP) (N = 58).

Variables, OR ^#^ (95% CI)	Null Model	Model with Level 1 Variables, OR (95% CI)	Model with Level 1 and Level 2 Variables, OR (95% CI)
Fixed-effects variables, varying within cluster			
		Patients’ characteristics (Level 1 variables)	
Gender Male Female		Reference 0.957 (0.869–1.053)	Reference 0.958 (0.870–1.055)
Age, year		0.981 (0.979–0.984) **	0.981 (0.979–0.984) **
Mode of transportation: Self-referral By ambulance		Reference 0.347 (0.287–0.420) **	Reference 0.345 (0.285–0.418) **
Triage severity scale			
2		Reference	Reference
3		4.515 (3.772–5.404) **	4.508 (3.766–5.397) **
4		4.156 (3.347–5.136) **	4.141 (3.342–5.132) **
Pain by numeric rating scale, unit		1.521 (1.488–1.554) **	1.521 (1.488–1.554) **
		Triage process and crowding	
Triage duration in minutes		1.083 (1.065–1.102) **	1.082 (1.064–1.101) **
Patients in waiting room, n		1.023 (1.012–1.035) **	1.023 (1.012–1.035) **
		Nurses’ characteristics (Level 2 variables)	
Gender Male Female			Reference 1.111 (0.763–1.617)
Age, year			1.000 (0.943–1.061)
Postgraduate experience, year			1.050 (0.959–1.148)
ED experience, year			0.934 (0.857–1.019)
Certification in emergency care No Yes			Reference 1.004 (0.620–1.625)
Risk-Taking Scale, points			1.068 (0.999–1.143)
Stress from Uncertainty Scale, points			1.023 (0.987–1.060)
Country of training Switzerland/Canada European Union			Reference 1.524 (1.038–2.237) *
Burden of prescribing responsibility No Yes			Reference 1.104 (0.703–1.736)
Number of different causes of severe pain, n (range, 0–6)			0.967 (0.852–1.096)
Fixed-effects variables, constant within cluster, IOR ^Φ^ (95% CI)			
Nurse gender Male Female			Reference 0.402–3.067
Nurses’ age			0.362–2.762
Certification in emergency care			0.364–2.771
Country of training, Switzerland/Canada European Union			Reference 0.552–4.207
Postgraduate experience			0.380–2.898
ED experience			0.338–2.579
Risk-Taking Scale			0.387–2.950
Stress from Uncertainty Scale			0.370–2.824
Burden of prescribing responsibility No Yes			Reference 0.400–3.049
Number of painful body areas			0.350–2.668
Random effects Nurse’s MOR ^Σ^	1.71	1.83	1.71
Log likelihood	−9055.6		−5231.8
N	11,228	11,228	11,228
Likelihood-ratio test	-	<0.001	<0.001

* *p* < 0.05; ** *p* < 0.001;. ^#^ OR = odds ratios; ^Φ^ IOR = interval odds ratios; ^Σ^ MOR = median odds ratios; CI = confidence interval. Logistic regression adjusted for means of level 1 variables. Complete-case analysis, using only encounters with no missing data and resulting in a reduced sample size.

## Data Availability

Data are available from the last author upon reasonable request.

## Supplementary Materials

The following supporting information can be downloaded at: https://www.mdpi.com/article/10.3390/jcm15020782/s1. Figure S1: Nurse-initiated pain protocol (NIPP); Supplementary S1: Analysis interpretation.
