# Supplementary material for: Impact of Patients, Nurses, and Workload on the Use of a Nurse-Initiated Pain Protocol at Triage in the Emergency Department: A Single-Center Retrospective Observational Study
Source: J Clin Med. 2026 Jan 18;15(2):782. doi: 10.3390/jcm15020782 (PMC12842159; doi:10.3390/jcm15020782)
Supplement: Supplementary file 1 [file jcm-15-00782-s001.zip › jcm-4056976-supplementary.pdf]

Journal of Clinical Medicine

MDPI

Supplementary Materials

Figure S1. Nurse-initiated pain protocol (NIPP).

NURSE-INITIATED PAIN PROTOCOL (NIPP) CHUV EMERGENCY DEPARTMENT

	Arrival<br>Non-pharmacological measures	Time 0<br>First pain assessment<br>First administration		Time 0 + 30 minutes<br>Second pain assessment<br>Second administration		Time 0 + 60 minutes<br>Third pain assessment<br>Third administration	
MUSCULOSKELETAL PAIN & RENAL COLIC	empathic attitude, pain-relieving position, immobilization, ice	NRS 1-3	Acetaminophen 1 g po	NRS 1-3	reassess pain in 30 minutes	NRS 1-3	reassess pain in 30 minutes
						NRS ≥ 4	**waiting room:** Ibuprofen 600 mg po<br>**treatment area:** MORPHINE PROTOCOL
				NRS ≥ 4	**waiting room:** Ibuprofen 600 mg po<br>**treatment area:** MORPHINE PROTOCOL	NRS 1-3	reassess pain in 30 minutes
						NRS ≥ 4	**waiting room:** Tramadol 50 mg po<br>**treatment area:** MORPHINE PROTOCOL
		NRS ≥ 4	**waiting room:**<br>Acetaminophen 1 g + Ibuprofen 600 mg po<br>**treatment area:**<br>Acetaminophen 1 g + MORPHINE PROTOCOL	NRS 1-3	reassess pain in 30 minutes	NRS 1-3	reassess pain in 30 minutes
						NRS ≥ 4	**waiting room:** Tramadol 50 mg po<br>**treatment area:** MORPHINE PROTOCOL
				NRS ≥ 4	**waiting room:** Tramadol 50 mg po<br>**treatment area:** MORPHINE PROTOCOL	NRS 1-3	reassess pain in 30 minutes
						NRS ≥ 4	evaluation by physician
ABDOMINAL PAIN	empathic attitude	NRS 1-3	Acetaminophen 1 g po	NRS 1-3	reassess pain in 30 minutes	NRS 1-3	reassess pain in 30 minutes
						NRS ≥ 4	**waiting room:** Tramadol 50 mg po<br>**treatment area:** MORPHINE PROTOCOL
				NRS ≥ 4	**waiting room :** Tramadol 50 mg po<br>**treatment area :** MORPHINE PROTOCOL	NRS 1-3	reassess pain in 30 minutes
						NRS ≥ 4	evaluation by physician
		NRS ≥ 4	**waiting room:**<br>Acetaminophen 1 g + Tramadol 50 mg po<br>**treatment area:**<br>Acetaminophen 1 g + MORPHINE PROTOCOL	NRS 1-3	reassess pain in 30 minutes	NRS 1-3	reassess pain in 30 minutes
						NRS ≥ 4	**call a physician or rapid transfer to treatment area:** MORPHINE PROTOCOL
				NRS ≥ 4	**call a physician or [rapid transfer to treatment area** MORPHINE PROTOCOL**]**	NRS 1-3	reassess pain in 30 minutes
						NRS ≥ 4	evaluation by physician
HEADACHE	empathic attitude, reduced sensory stimuli (light, noise)	NRS 1-3	Acetaminophen 1 g po	NRS 1-3	reassess pain in 30 minutes	CONTRINDICATIONS TO NIPP USE	
				NRS ≥ 4	**call a physician** for Ibuprofen 600 mg po **or rapid transfer to treatment area**		
		NRS ≥ 4	Acetaminophen 1 g po + **call a physician** for Ibuprofen 600 mg po **or rapid transfer to treatment area**	NRS 1-3	reassess pain in 30 minutes	**Acute alcohol intoxication, drug abuse, chronic pain (> 3 months), drug abuse, children up to 16 ⇒ NO analgesic administration without physician's assessment**	
				NRS ≥ 4	**re-evaluation by physician**		

Contraindications to acetaminophen	Contraindications to ibuprofen	Contraindications to tramadol
Liver failure, allergy, taking 1 g in the previous 4 hours or 4 g in the previous 24 hours	Allergy to aspirin/NSAIDs, NSAIDs taken within the previous 6 hours, pregnancy, epigastralgia, peptic ulcer disease, renal, cardiac or hepatic failure	Pregnancy 0-15 weeks, allergy, epilepsy, respiratory failure (COPD or asthma attack), hepatic or renal failure, taking 50 mg within the previous 6 hours or 200 mg within 12 hours

If contraindication to one of these drugs → Call by the triage nurse to ED physician to prescribe an alternative analgesic treatment, or if previously taking a drug at a lower dose than that of protocol, complete the dose and/or molecule according to the NIPP

Supplementary S1. Analysis interpretation.

For patient variables (level 1), the interpretation of the model coefficients is similar to that of standard logistic regression, i.e. adjusted log odds ratios (logORs), except that they must be interpreted as subject-specific effects, unlike classical logistic regression analysis where the coefficient of a co-variable is interpreted as a population logOR. For cluster-level 2 variables (i.e. nurse characteristics), the model coefficients should be interpreted as median logORs, since ORs at level 2 are not fixed any longer but random, and it is necessary to compare nurses with different random effects [1].

The residual effect of context (i.e. the effect of nurse clusters on the outcome after accounting for nurse-level variables) was quantified using the median OR (MOR), whereas the interval OR (IOR) was used when assessing the association between context variables (i.e. nurse characteristics) and the outcome [2]. In brief, the MOR allows to quantify a residual context effect on the same scale as individual variables (i.e. the OR) and is therefore more intuitive than the intra-class correlation (ICC). The MOR represents the median increase of the outcome for a patient switched from a cluster with a lower to a cluster with higher odds, all the other variables remaining constant [3]. On the other hand, the IOR reflects the variation of the OR due to the random effect in the linear predictor, i.e. the IOR evaluates the magnitude of context residual variations when interpreting the

effect of context-level variables. The IOR is narrow if the residual variation between clusters is small and large otherwise. An IOR containing the value 1 indicates that the effect of a specific cluster variable is weak in comparison with the residual heterogeneity between clusters [4].

1. Larsen, K.; Petersen, J.H.; Budtz-Jorgensen, E.; Endahl, L. Interpreting parameters in the logistic regression model with random effects. *Biometrics* **2000**, *56*, 909-914, doi:10.1111/j.0006-341x.2000.00909.x.
2. Larsen, K.; Merlo, J. Appropriate assessment of neighborhood effects on individual health: integrating random and fixed effects in multilevel logistic regression. *Am J Epidemiol* **2005**, *161*, 81-88, doi:10.1093/aje/kwi017.
3. Yarnell, C.; Pinto, R.; Fowler, R. Measuring variability between clusters by subgroup: An extension of the median odds ratio. *Stat Med* **2019**, *38*, 4253-4263, doi:10.1002/sim.8286.
4. Merlo, J.; Chaix, B.; Ohlsson, H.; Beckman, A.; Johnell, K.; Hjerpe, P.; Rastam, L.; Larsen, K. A brief conceptual tutorial of multilevel analysis in social epidemiology: using measures of clustering in multilevel logistic regression to investigate contextual phenomena. *J Epidemiol Community Health* **2006**, *60*, 290-297, doi:10.1136/jech.2004.029454.

https://doi.org/10.3390/jcm15020782
